# Influence of NOS3 rs2070744 genotypes on hepatocellular carcinoma patients treated with lenvatinib

**DOI:** 10.1038/s41598-020-73930-3

**Published:** 2020-10-13

**Authors:** Shintaro Azuma, Haruki Uojima, Makoto Chuma, Xue Shao, Hisashi Hidaka, Takahide Nakazawa, Masaaki Kondo, Kazushi Numata, Shogo Iwabuchi, Makoto Kako, Shin Maeda, Wasaburo Koizumi, Koichiro Atsuda

**Affiliations:** 1 Department of Pharmacy, Kitasato University Hospital, 1-15-1 Kitasato, Minami-ku, Sagamihara, Kanagawa 252-0375 Japan; 2 Graduate School of Pharmaceutical Sciences, Kitasato University, 5-9-1 Shirokane, Minato-ku, Tokyo, 108-8641 Japan; 3 Department of Gastroenterology, Internal Medicine, Kitasato University School of Medicine, 1-15-1 Kitasato, Minami-ku, Sagamihara, Kanagawa 252-0375 Japan; 4 Gastroenterology Medicine Center, Shonan Kamakura General Hospital, 1370-1 Okamoto, Kamakura, Kanagawa 247-8533 Japan; 5 Gastroenterological Center, Yokohama City University Medical Center, 4-57 Urahune, Minami-ku, Yokohama, Kanagawa 232-0024 Japan; 6 Nakazawa Internal Medicine Clinic, 4-14-18 Sagamidai, Minami-ku, Sagamihara, Kanagawa 252-0321 Japan; 7 Department of Gastroenterology, Yokohama City University Hospital, 3-9 Fukuura, Kanazawa-ku, Yokohama, Kanagawa 236-0004 Japan; 8 Hepato-Biliary-Pancreatic Center, Shonan Fujisawa Tokushukai Hospital, 1-5-1 Tsujidokandai, Fujisawa, Kanagawa 251-0041 Japan; 9 School of Pharmaceutical Sciences, Kitasato University, 5-9-1 Shirokane, Minato-ku, Tokyo, 108-8641 Japan

**Keywords:** Cancer, Genetics, Biomarkers, Gastroenterology, Medical research, Molecular medicine, Oncology

## Abstract

We investigated whether or not nitric oxide synthase 3 (NOS3) rs2070744 genotypes can affect the response for lenvatinib treatment in patients with hepatocellular carcinoma (HCC). We evaluated the relation of the NOS3 rs2070744 genotypes to the tumor response, progression-free survival (PFS), and overall survival (OS) as the response for lenvatinib. We also examined the association between fibroblast growth factor receptor (FGFR) gene polymorphisms, a potential feature of lenvatinib, and the response. There were no significant differences between the studies for either PFS or OS, even though patients with the TT genotype had a longer mean PFS (hazard ratio [HR] 0.60; *p* = 0.069) and mean OS (HR 0.46; *p* = 0.075) than those with the TC/CC genotypes. However, patients with a single-nucleotide polymorphism (SNP) combination pattern of the NOS3 rs2070744 TC/CC and FGFR4 rs351855 CT/TT genotypes had a significantly shorter mean PFS (HR 2.56; *p* = 0.006) and mean OS (HR 3.36; *p* = 0.013) than those with the other genotypes. The NOS3 rs2070744 genotypes did not influence the clinical response. However, the SNP combination pattern of the NOS3 rs2070744 and FGFR4 rs351855 genotypes may be helpful as treatment effect predictors and prognostic factors for HCC patients treated with lenvatinib.

## Introduction

Lenvatinib, a novel multikinase inhibitor, was found to be comparable to sorafenib in overall survival (OS) in unresectable hepatocellular carcinoma (HCC) patients in a randomized phase III non-inferiority (REFLECT) trial^1–5^. Therefore, it is now prescribed as the first-line therapy for advanced unresectable HCC. Compared with those for cancer-targeted treatments, the predictive factors for the response to lenvatinib, which target the microenvironment of the tumor, are unknown. The identification of such markers could improve the management of HCC by selecting the most effective therapeutic options available^6^. Some researchers have reported that a single-nucleotide polymorphism (SNP) of the endothelial nitric oxide synthase (eNOS) or the *NOS3* gene influences the response to sorafenib in patients with HCC^7,8^. The production of endogenous NO plays an essential role in carcinogenesis and tumor growth progression.

Three distinct genes, *NOS1*, *NOS2*, and *NOS3*, encode the NOS isoforms: the neuronal (nNOS), inducible (iNOS), and eNOS, and the genetic variations present in genes encoding for NOS isoforms can affect NOS expression^9^. A point mutation of thymine to cytosine at the NOS3 T-786C polymorphism (rs2070744) results in the altered NOS protein levels and enzymatic activity^10,11^. The previous study has reported a correlation between the NOS3 rs2070744 genotypes and the clinical response to sorafenib, which exerts antitumor effects by inhibiting vascular endothelial growth factor receptor (VEGFR), in patients with HCC^12^.

Lenvatinib exerts antitumor effects by inhibiting receptor tyrosine kinases, including VEGFR^5^. The NOS3 rs2070744 genotypes may be useful predictors of the clinical response to lenvatinib. However, lenvatinib is a multiple receptor tyrosine kinase inhibitor targeting not only VEGFR but also fibroblast growth factor receptor (FGFR), platelet-derived growth factor receptor alpha, and KIT and RET proto-oncogenes. The potent activity against FGFR is a characteristic of lenvatinib, unlike sorafenib. Thus, there may be a difference in the responsiveness of NOS3 polymorphisms to HCC between lenvatinib and sorafenib. Therefore, we analyzed the role of the NOS3 polymorphism on HCC patients treated with lenvatinib.

## Results

### Enrolled patients

Of the 168 patients enrolled in this study, 68 (40.5%) patients were excluded (see Supplementary Fig. S1 online). Therefore, relevant clinical data were collected from 100 patients in three medical centers in Kanagawa: Kitasato University Hospital (n = 56), Yokohama City University Medical Center (n = 27), and Shonan Kamakura General Hospital (n = 17). All the patients from Shonan Fujisawa Tokushukai Hospital were excluded from the study because they did not meet the inclusion criteria.

### Correlation between the NOS3 rs2070744 genotypes and clinical characteristics

The demographic characteristics of patients are summarized in Table 1. The frequencies of TT, TC, and CC of the NOS3 rs2070744 genotypes were 76%, 23%, and 1%, respectively. The number of patients with the TT and TC/CC genotypes was 76 and 24, respectively. Comparing the clinical characteristics of patients with the TT genotype with those of patients with the TC/CC genotypes, there were no significant differences between the groups.

**Table 1:** Baseline clinical characteristics.

Characteristics	All (N = 100)	NOS3 rs2070744		*p*
		TT (n = 76)	TC/CC (n = 24)	
**Median age (IQR), years**	72 (66–78)	73 (66–79)	72 (65–78)	0.625
**Age ≥ 65 years, n (%)**	76 (76)	58 (76)	18 (75)	1.000
**Age ≥ 75 years, n (%)**	43 (43)	35 (46)	8 (33)	0.347
**Male sex, n (%)**	68 (68)	54 (71)	14 (58)	0.316
**Etiology of HCC, n (%)**				
Hepatitis B	17 (17)	12 (16)	5 (21)	0.504
Hepatitis C	41 (41)	34 (45)	7 (29)	
Alcohol use	15 (15)	10 (13)	5 (21)	
Non-alcoholic steatohepatitis	21 (21)	14 (18)	7 (29)	
Others	7 (7)	6 (8)	1 (4)	
**ECOG-PS, n (%)**				
0	95 (95)	71 (93)	24 (100)	0.333
1	5 (5)	5 (7)	0	
**Child–Pugh system, n (%)**				
Class A5	61 (61)	46 (61)	15 (63)	0.984
Class A6	22 (22)	17 (22)	5 (21)	
Class B	17 (17)	13 (17)	4 (17)	
**Bodyweight ≥ 60 kg, n (%)**	59 (59)	44 (58)	15 (63)	0.813
**Median BMI (IQR), kg/m**^**2**^	23.0 (21.1–25.2)	22.9 (21.1–25.1)	23.2 (20.8–26.7)	0.663
**BCLC staging system, n (%)**				
Stage B (intermediate stage)	55 (55)	41 (54)	14 (58)	0.815
Stage C (advanced stage)	45 (45)	35 (46)	10 (42)	
**Macroscopic PVI, extrahepatic spread, or both, present, n (%)**	37 (37)	27 (36)	10 (42)	0.303
**Up-to-seven criteria, n (%)**				
within	60 (60)	45 (59)	15 (63)	0.816
beyond	40 (40)	31 (41)	9 (38)	
**Maximum tumor size > 5 cm, n (%)**	28 (28)	22 (29)	6 (25)	0.799
**Number of tumors, n (%)**				
1	14 (14)	10 (13)	4 (17)	0.703
2	13 (13)	11 (14)	2 (8)	
≥ 3	69 (69)	52 (68)	17 (71)	
**UICC-TNM staging system, n (%)**				
I and II	25 (25)	18 (24)	7 (29)	0.597
III and IV	75 (75)	58 (76)	17 (71)	
**Tumor liver occupying rate ≥ 50%, n (%)**	6 (6)	4 (5)	2 (8)	0.628
**Initial dose, n (%)**				
12 mg	32 (32)	23 (30)	9 (38)	0.704
8 mg	49 (49)	39 (51)	10 (42)	
4 mg	19 (19)	14 (18)	5 (21)	
**RDI at 56 days ≥ 80%, n (%)**	44 (44)	31 (41)	13 (54)	0.328
**ALBI grade**				
Grade 1 (≤ − 2.60)	44 (44)	36 (47)	8 (33)	0.372
Grade 2 (> − 2.60 to ≤ − 1.39)	54 (54)	39 (51)	15 (63)	
Grade 3 (> − 1.39)	2 (2)	1 (1)	1 (4)	
**AFP at baseline ≥ 200 ng/mL, n (%)**	35 (35)	24 (32)	11 (46)	0.226
**AFP at baseline ≥ 400 ng/mL, n (%)**	31 (31)	22 (29)	9 (38)	0.455
**Median PIVKA- II (IQR), mAU/mL**	392 (74–2200)	346 (74–2420)	672 (105–1489)	0.686

*AFP* α-fetoprotein, *ALBI* albumin-bilirubin, *BCLC* Barcelona Clinic Liver Cancer, *BMI* body mass index, *ECOG-PS* Eastern Cooperative Oncology Group-Performance Status, *HCC* hepatocellular carcinoma, *IQR* interquartile range, *NOS3* nitric oxide synthase 3, *PIVKA-II *protein induced by vitamin K absence or antagonist II, *PVI* portal vein invasion, *RDI* relative dose intensity.

### Effect of NOS3 rs2070744 on tumor response

Tumor response rates are summarized in Table 2. The confirmed objective response rates were 54% with the NOS3 rs2070744 TC/CC genotypes and 42% with the TT genotype (odds ratio [OR] 1.63; 95% confidence interval [CI] 0.58–4.56; *p* = 0.351). The disease control rate (objective response plus stable disease) was 83% with the TC/CC genotypes and 84% with the TT genotype (OR 0.94; 95% CI 0.25–4.44; *p* = 1.000).

**Table 2:** Effect of the NOS3 rs2070744 on tumor response, progression-free survival, and overall survival.

Variable	N	Tumor response						Progression-free survival			Overall survival		
		ORR, n (%)	OR (95% CI)	*p*	DCR, n (%)	OR (95% CI)	*p*	Mean months (95% CI)	HR (95% CI)	*p*	Mean months (95% CI)	HR (95% CI)	*p*
**NOS3 rs2070744**													
TC + CC	24	13 (54)	1.63 (0.58–4.56)	0.351	20 (83)	0.94 (0.25–4.44)	1.000	7.5 (4.8–10.2)	1 (ref)	0.069	16.1 (12.7–19.4)	1 (ref)	0.075
TT	76	32 (42)			64 (84)			10.6 (8.9–12.4)	0.60 (0.34–1.05)		22.3 (20.2–24.4)	0.46 (0.19–1.11)	

*CI* confidence interval, *DCR* disease control rate (objective response plus stable disease), *HR* hazard ratio, *NOS3* nitric oxide synthase 3, *OR* odds ratio, *ORR* objective response rate (complete and partial response).

### Effect of NOS3 rs2070744 on PFS and OS

**Figure 1:**
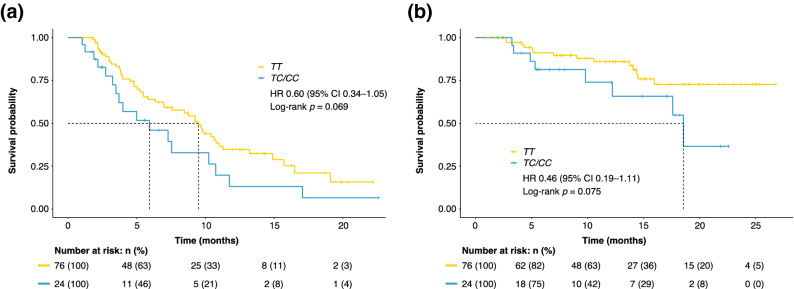
(**a**) Kaplan–Meier curves of PFS according to the NOS3 rs2070744 genotypes: TC/CC (blue line) vs. TT (yellow line). (**b**) Kaplan–Meier curves of OS according to the NOS3 rs2070744 genotypes: TC/CC (blue line) vs. TT (yellow line). *CI* confidence interval, *HR* hazard ratio, *NOS3* nitric oxide synthase 3, *OS* overall survival, *PFS* progression-free survival.

Figure 1 and Table 2 show the effect of the NOS3 rs2070744 genotypes on clinical outcomes. The mean progression-free survival (PFS) of all enrolled patients was 10.0 (95% CI 8.5–11.5) months. The mean PFS of the NOS3 rs2070744 TC/CC genotypes and the TT genotype was 7.5 (95% CI 4.8–10.2) months and 10.6 (95% CI 8.9–12.4) months, respectively. There were no significant differences between the PFS of the two groups (HR 0.60; 95% CI 0.34–1.05; *p* = 0.069). The mean OS of all enrolled patients was 21.3 (95% CI 19.4–23.3) months. The mean OS of the TC/CC genotypes and TT genotype was 16.1 (95% CI 12.7–19.4) months and 22.3 (95% CI 20.2–24.4) months, respectively. There were no significant differences between the OS of the two groups (HR 0.46; 95% CI 0.19–1.11; *p* = 0.075).

### Effects of the *FGFR* gene polymorphisms on clinical response

Table 3 shows the effect of the *FGFR* gene polymorphisms on clinical outcomes. FGFR2 (rs2912791, rs2981429, rs2981582) and FGFR4 (rs351855) showed no significant differences in tumor response, PFS, or OS.

**Table 3:** Effects of FGFR alone and in combination with NOS3 polymorphism on tumor response, progression-free survival, and overall survival.

Variable	N	Tumor response						Progression-free survival			Overall survival		
		ORR, n (%)	OR (95% CI)	*p*	DCR, n (%)	OR (95% CI)	*p*	Mean months (95% CI)	HR (95% CI)	*p*	Mean months (95% CI)	HR (95% CI)	*p*
**FGFR2 rs2912791**													
CT + CC	66	31 (47)	0.79 (0.31–1.97)	0.673	57 (86)	0.61 (0.18–2.16)	0.398	10.4 (8.5–12.3)	1 (ref)	0.490	21.5 (19.1–23.8)	1 (ref)	0.771
TT	34	14 (41)			27 (79)			9.3 (6.8–11.8)	1.20 (0.72–1.99)		19.8 (16.6–22.9)	1.14 (0.48–2.71)	
**FGFR2 rs2981429**													
CT + TT	48	23 (48)	0.80 (0.34–1.89)	0.688	41 (85)	0.82 (0.23–2.73)	0.789	10.4 (8.2–12.5)	1 (ref)	0.541	21.5 (18.9–24.1)	1 (ref)	0.742
CC	52	22 (42)			43 (83)			9.5 (7.3–11.6)	1.17 (0.71–1.91)		19.9 (17.3–22.5)	1.15 (0.50–2.66)	
**FGFR2 rs2981582**													
CT + CC	91	41 (45)	0.98 (0.18–4.86)	1.000	77 (85)	0.64 (0.11–6.93)	0.633	10.0 (8.4–11.7)	1 (ref)	0.817	21.0 (19.0–23.1)	1 (ref)	0.441
TT	9	4 (44)			7 (78)			10.4 (7.4–13.4)	0.91 (0.39–2.11)		21.1 (15.5–26.6)	0.46 (0.06–3.45)	
**FGFR4 rs351855**													
CT + TT	63	28 (44)	0.94 (0.39–2.31)	1.000	54 (86)	1.40 (0.40–4.71)	0.580	10.3 (8.4–12.2)	1 (ref)	0.552	21.4 (19.0–23.8)	1 (ref)	0.963
CC	37	17 (46)			30 (81)			9.3 (6.9–11.7)	1.17 (0.70–1.94)		19.8 (16.9–22.6)	1.02 (0.43–2.44)	
**NOS3 rs2070744 + FGFR4 rs351855**													
Others	87	41 (47)	0.50 (0.10–1.97)	0.373	74 (85)	0.59 (0.13–3.77)	0.432	10.6 (9.0–12.3)	1 (ref)	0.006	22.0 (20.1–24.0)	1 (ref)	0.013
TC/CC + CT/TT	13	4 (31)			10 (77)			6.0 (2.7–9.3)	2.56 (1.28–5.09)		12.0 (8.1–15.8)	3.36 (1.21–9.29)	

*CI* confidence interval, *DCR* disease control rate (objective response plus stable disease), *FGFR2* fibroblast growth factor receptor 2, *FGFR4* fibroblast growth factor receptor 4, *HR* hazard ratio, *NOS3* nitric oxide synthase 3, *OR* odds ratio, *ORR* objective response rate (complete and partial response).

### The SNP combination pattern and clinical outcomes

**Figure 2:**
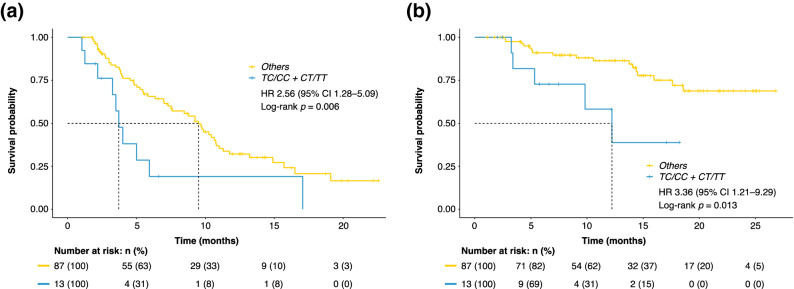
(**a**) Kaplan–Meier curves of PFS according to an SNP combination pattern of the NOS3 rs2070744 and FGFR4 rs351855: TC/CC + CT/TT genotypes (blue line) vs. Others (yellow line). (**b**) Kaplan–Meier curves of OS according to an SNP combination pattern of the NOS3 rs2070744 and FGFR4 rs351855: TC/CC + CT/TT genotypes (blue line) vs. Others (yellow line). *CI* confidence interval, *FGFR4* fibroblast growth factor receptor 4, *HR* hazard ratio, *NOS3* nitric oxide synthase 3, *OS* overall survival, *PFS* progression-free survival, *SNP* single-nucleotide polymorphism.

Figure 2 and Table 3 show the effect of FGFR alone and in combination with the NOS3 polymorphism on clinical outcomes. According to the effects of an SNP combination pattern of the NOS3 rs2070744 and FGFR4 rs351855 genotypes, patients with the NOS3 rs2070744 TC/CC and FGFR4 rs351855 CT/TT genotypes had a significantly shorter mean PFS of 6.0 (95% CI 2.7–9.3) months than did those with the other genotypes who had a mean PFS of 10.6 (95% CI 9.0–12.3) months (HR 2.56; 95% CI 1.28–5.09; *p* = 0.006). Similarly, patients with the TC/CC and CT/TT genotypes had a significantly shorter mean OS of 12.0 (95% CI 8.1–15.8) months than did those with the other genotypes who had a mean OS of 22.0 (95% CI 20.1–24.0) months (HR 3.36; 95% CI 1.21–9.29; *p* = 0.013).

### Univariate and multivariate analysis of factors affecting the PFS and OS

We evaluated the relationship between the clinical response and the baseline clinical characteristics using a logistic regression model (see Supplementary Table S1 online). Table 4 provides the factors most associated with survival. In multivariate analysis adjusted for the Child–Pugh system (Class A vs. Class B), α-fetoprotein (< 400 vs. ≥ 400), age (< 75 vs. ≥ 75), albumin-bilirubin (ALBI) grade (Grade 1 vs. Grade ≥ 2), Barcelona Clinic Liver Cancer (BCLC) staging system (Stage B vs. Stage C), Macroscopic portal vein invasion, extrahepatic spread, or both, present (no vs. yes), and relative dose intensities at 56 days (≥ 80% vs. < 80%). The SNP combination pattern of the NOS3 rs2070744 TC/CC and FGFR4 rs351855 CT/TT genotypes was significantly associated with both PFS (HR 2.95; 95% CI 1.27–6.86; *p* = 0.012) and OS (HR 3.29; 95% CI 1.01–10.74; *p* = 0.049), respectively.

**Table 4:**
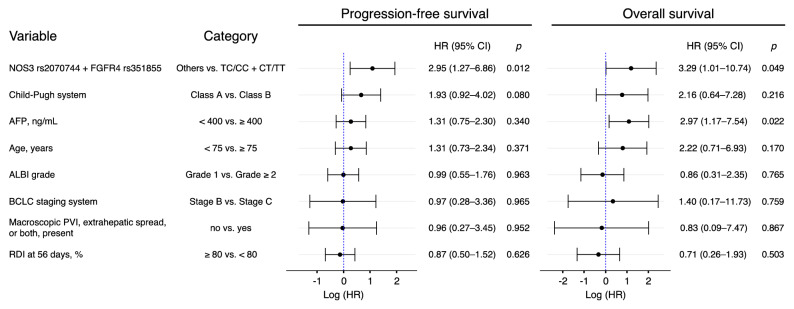
Multivariable analysis for baseline prognostic factors for progression-free survival and overall survival.

*AFP* α-fetoprotein, *ALBI* albumin-bilirubin, *BCLC* Barcelona Clinic Liver Cancer, *CI* confidence interval, *FGFR4* fibroblast growth factor receptor 4, *HR* hazard ratio, *NOS3* nitric oxide synthase 3, *PVI* portal vein invasion, *RDI* relative dose intensity.

### Adverse event assessment

The most common adverse events among patients who received lenvatinib were anorexia (59%), fatigue (55%), palmar-plantar erythrodysesthesia (47%), abdominal pain (41%), and diarrhea (41%) (see Supplementary Table S2 online). Adverse events associated with lenvatinib forced dose reductions and discontinuation in 57% and 46% of the patients, respectively. The median time to the first dose reduction was 1.3 (interquartile range [IQR]; 0.8–3.0) months among the patients for whom the dose was reduced due to adverse events. The median time to dose reduction of the NOS3 rs2070744 TT genotype and TC/CC genotypes was 1.3 (IQR; 0.8–3.0) months and 1.4 (IQR; 0.9–3.4) months, respectively. There were no significant differences between the median time of the two groups (*p* = 0.627). Grade 3 adverse events occurred in 33 (43%) patients with the NOS3 rs2070744 TT genotype and 10 (42%) patients with the TC/CC genotypes. Grade 3 adverse events also occurred in 16 (43%) patients with the FGFR4 rs351855 CC genotype and 27 (43%) patients with the CT/TT genotypes. No grade 4 adverse events were observed. The median treatment duration was 6.4 (IQR; 2.5–12.6) months for the enrolled patients. The median treatment duration with the NOS3 rs2070744 TT genotype was longer than that with the TC/CC genotypes (6.9 [IQR; 2.5–12.9] months vs. 5.6 [IQR; 2.4–9.0] months,* p* = 0.611); however, the incidence rate of adverse events was similar.

Table 5 shows the impact on clinical outcomes of adverse events with an incidence rate of 30% or more. The mean PFS of the patients who developed grades 2 or 3 hypertension (n = 20) was significantly longer than that for those who developed grade 1 hypertension (n = 15) (10.9 [95% CI 7.5–14.2] months vs. 5.5 [95% CI 3.1–7.8] months; HR 0.37 [95% CI 0.17–0.83]; *p* = 0.012). There were no significant differences between the OS of the two groups (22.7 [95% CI 19.1–26.3] months vs. 16.6 [95% CI 11.3–21.9] months; HR 0.38 [95% CI 0.11–1.36]; *p* = 0.123). There were no correlations between the genetic polymorphisms, including those of the *NOS3* gene, and the prevalence and severity of adverse events.

**Table 5:** Effect of the adverse events on progression-free survival and overall survival.

Variable	N	Progression-free survival			Overall survival		
		Mean months (95% CI)	HR (95% CI)	*p*	Mean months (95% CI)	HR (95% CI)	*p*
**Anorexia**							
Grade 1	10	8.8 (3.7–13.9)	1 (ref)	0.702	26.8 (26.8–26.8)	N/A	0.087
Grade ≥ 2	49	8.7 (6.8–10.5)	1.19 (0.50–2.84)		18.8 (16.2–21.4)		
**Fatigue**							
Grade 1	31	8.6 (6.3–11.0)	1 (ref)	0.540	19.9 (16.6–23.2)	1 (ref)	0.429
Grade ≥ 2	24	7.6 (5.4–9.7)	1.21 (0.66–2.22)		18.6 (14.7–22.5)	1.48 (0.56–3.93)	
**PPE**							
Grade 1	25	8.3 (5.9–10.8)	1 (ref)	0.547	21.1 (17.3–25.0)	1 (ref)	0.857
Grade ≥ 2	22	9.5 (6.6–12.5)	0.82 (0.42–1.58)		19.7 (16.1–23.2)	1.11 (0.37–3.31)	
**Abdominal pain**							
Grade 1	26	8.3 (5.5–11.0)	1 (ref)	0.779	18.9 (15.3–22.4)	1 (ref)	0.929
Grade ≥ 2	15	7.8 (5.1–10.5)	1.10 (0.55–2.21)		20.1 (15.2–25.0)	0.95 (0.31–2.91)	
**Diarrhea**							
Grade 1	24	7.1 (5.3–8.9)	1 (ref)	0.201	17.5 (13.7–21.3)	1 (ref)	0.154
Grade ≥ 2	17	10.7 (6.5–14.9)	0.62 (0.29–1.30)		21.3 (17.7–24.9)	0.40 (0.11–1.48)	
**Dysgeusia**							
Grade 1	29	7.3 (5.6–9.0)	1 (ref)	0.958	19.6 (16.0–23.1)	1 (ref)	0.635
Grade ≥ 2	7	7.0 (4.2–9.8)	1.02 (0.42–2.52)		16.5 (9.3–23.7)	1.37 (0.38–4.97)	
**Edema**							
Grade 1	24	9.9 (6.9–12.8)	1 (ref)	0.584	24.8 (22.1–27.5)	1 (ref)	0.137
Grade ≥ 2	12	9.0 (5.7–12.2)	1.24 (0.57–2.70)		19.7 (15.1–24.4)	3.38 (0.62–18.6)	
**Anxiety**							
Grade 1	28	7.2 (5.4–9.1)	1 (ref)	0.447	19.6 (16.3–22.9)	1 (ref)	0.449
Grade ≥ 2	7	8.9 (3.6–14.1)	0.69 (0.26–1.82)		16.7 (9.5–24.0)	1.66 (0.44–6.29)	
**Hypertension**							
Grade 1	15	5.5 (3.1–7.8)	1 (ref)	0.012	16.6 (11.3–21.9)	1 (ref)	0.123
Grade ≥ 2	20	10.9 (7.5–14.2)	0.37 (0.17–0.83)		22.7 (19.1–26.3)	0.38 (0.11–1.36)	

*CI* confidence interval, *HR* hazard ratio, *N/A* not available, *PPE* palmar-plantar erythrodysesthesia.

## Discussion

To the best of our knowledge, this is the first study to report a correlation between the NOS3 rs2070744 genotypes and the clinical response to lenvatinib in patients with unresectable HCC. In a study investigating the utility of NOS3 SNPs, the NOS3 rs2070744 TT genotype was strongly associated with worse prognoses in patients with advanced HCC treated with sorafenib^12^. However, the NOS3 rs2070744 TC/CC genotypes tended to correspond to a worse prognosis after the administration of lenvatinib, although there were no statistically significant differences. That is, there was a difference in the responsiveness of NOS3 polymorphisms to HCC between the administration of lenvatinib and sorafenib. This can be attributed to the fact that lenvatinib is a multiple receptor tyrosine kinase inhibitor targeting not only VEGFR but also FGFR.

HCC is a hypervascularized tumor. Tumor angiogenesis can be effectively suppressed by simultaneously suppressing both VEGFR and FGFR signals. Lenvatinib inhibits angiogenic factor-mediated pathways, including VEGF and FGF, resulting in a subsequent further reduction in NO production. Reduced production of NO, a vasodilator, induces contraction of hepatic stellate cells^13^. Moreover, it increases intrahepatic vascular resistance and decreases intrahepatic blood flow as well. This infers that the portal hemodynamic effects of lenvatinib are different from those of sorafenib. Lenvatinib significantly aggravates the Congestion index (− 23.1% vs. + 16.2%), which reflects portal hemodynamics, and may exacerbate portal hypertension in patients with advanced HCC^14,15^.

In the present study, we also revealed that an SNP combination pattern of the NOS3 rs2070744 and FGFR4 rs351855 genotypes was associated with PFS and OS. In another study, it was suggested that the FGFR4 Gly388Arg polymorphism (rs351855) resulted in the transmembrane domain missense mutation and may affect protein expression^16^. Mutations in the transmembrane domain of the tyrosine kinase receptor can alter the pathological phenotype or signal transduction of FGFR4^17^. Because FGFR4 has the highest expression of transcripts in the liver, FGFR4 rs351855 may regulate HCC signaling^18–20^. Moreover, the FGFR4 rs351855 CT/TT genotypes sustained receptor activation and improved stability of the receptor more than did the CC genotype. This suggested influence on the response to chemotherapy in HCC patients treated with tyrosine kinase inhibitors^21–24^.

The NOS3 rs2070744 TC/CC genotypes may reduce the promoter activity of the *NOS3* gene compared to the TT genotype, resulting in reduced NO production^25–27^. As is the case with the portal hemodynamic study^14,15^, the effects of NO with the NOS3 rs2070744 when treated with lenvatinib may differ from the results of a previous study^12^. Therefore, we presumed that the NOS3 rs2070744 and FGFR4 rs351855 genotypes could be useful predictors of the clinical response to lenvatinib treatment. The results revealed that the SNP combination pattern of the NOS3 rs2070744 and FGFR4 rs351855 genotypes was helpful as treatment effect predictors and prognostic factors for HCC patients treated with lenvatinib. However, there was no significant difference in the objective response rate. The objective response rate can not always be used to directly evaluate the clinical benefit nor can it be comprehensively used to measure drug efficacy. More research is needed to validate whether or not this SNP combination pattern can be a potential biomarker for chemotherapy.

There have been cases reported correlating patients' clinical response to treatment with drug-related adverse events^28–30^. In this study, patients who developed grades 2 or 3 hypertension during lenvatinib treatment had significantly longer PFS than those who developed grade 1 hypertension. Moreover, palmar-plantar erythrodysesthesia and diarrhea of grades 2 or 3 showed a prolonged PFS compared with grade 1. Previous studies have shown that patients who experienced hypertension and hand-foot skin reaction during lenvatinib treatment had significantly better outcomes than did those who did not develop these adverse events^31,32^. Our findings indicate that hypertension can be a positive predictive marker of prognosis in patients receiving lenvatinib treatment. VEGFR inhibitors such as sorafenib and lenvatinib have been reported to cause endothelial dysfunction associated with reduced NO production and activation of endothelin-1, a potent vasoconstrictor^33–37^. However, it is unclear why hypertension develops and why it is a marker of improved prognosis. On the other hand, grades 2 or 3 of anorexia and/or fatigue were poor prognostic factors for survival, although the differences were not significant. Anorexia was reported to be one of the common adverse events and was associated with time to discontinuation of lenvatinib treatment^32^. Anorexia and fatigue may be challenging conditions to alleviate and may affect the continuation of treatment and quality of daily life. The impact of genetic polymorphisms on the prevalence and severity of adverse events should be assessed, and the mechanisms underlying the occurrence of adverse events should be elucidated.

In this study, 55 (55%) patients were in the intermediate BCLC stage B and received systemic lenvatinib therapy. The global standard of care recommended for intermediate BCLC stage B is transcatheter arterial chemoembolization (TACE)^38,39^. However, recent clinical studies have shown that lenvatinib treatment for patients with intermediate-stage HCC who present with beyond up-to-seven criteria and have Child–Pugh class A prolonged PFS and OS^40,41^. Therefore, patients in this study with intermediate BCLC stage B and thus less likely to benefit from TACE were also treated with lenvatinib from the initial step at the discretion of their physicians. This study can help provide evidence for the importance of lenvatinib as a first-line treatment in patients with intermediate-stage HCC, who are very difficult to manage with TACE alone.

There were three limitations to this study. First, the selection bias for enrolled patients in this study cohort was undeniable because genomic DNA could not be obtained from all the patients receiving lenvatinib. Some patients, who had a short survival after lenvatinib treatment, could not be enrolled in this study. Therefore, individuals who had an inadequate response to lenvatinib were less likely to participate in the study. The median survival time of all the enrolled patients in this study was longer than that in the REFLECT trial with lenvatinib vs. sorafenib as a first-line treatment in 2018^5^. Moreover, the selection bias was correlated with the outcome. The multivariate analysis showed that an SNP combination pattern of the NOS3 rs2070744 and FGFR4 rs351855 genotypes was an independent factor of lenvatinib response, and a previous report showed that the factors significantly associated with poor OS included skeletal muscle mass index and ALBI grade^42^. Second, there was no control arm in the present study. Therefore, a clear distinction could not be made between the prognostic role of the NOS3 rs2070744 genotypes in patients treated with lenvatinib. Finally, the expression levels of NOS, which are crucial for NO synthesis, were not evaluated. Therefore, NOS expression and NO levels warrant further study and elucidation in patients with HCC.

In conclusion, the NOS3 rs2070744 genotypes did not influence the clinical response. However, we found that the SNP combination pattern of the NOS3 rs2070744 and FGFR4 rs351855 genotypes may be helpful as treatment effect predictors and prognostic factors for HCC patients treated with lenvatinib. Multicenter and prospective studies enrolling an appropriate number of patients will be required to confirm further the correlation of genetic polymorphisms with clinical outcomes and adverse events of lenvatinib.

## Methods

### Ethics

The Institutional Review Board (Ethics Committee) approved this study at all study the institutes involved, and the protocol of this study conforms to the provisions of the Declaration of Helsinki. This study is registered in the University Hospital Medical Information Network (UMIN) Clinical Trials Registry (UMIN000036625).

The study was approved by the research ethics board at each participating center, and written informed consent was obtained from all participants. Patients were informed about the risk and benefits of the research and then chose to give their consent to participate.

### Patients

This retrospective multicenter study was conducted in four medical institutions: Kitasato University Hospital, Yokohama City University Medical Center, Shonan Kamakura General Hospital, and Shonan Fujisawa Tokushukai Hospital in Japan.

Eligible patients (age, ≥ 20 years) with advanced or unresectable HCC caused by chronic liver disease, the Eastern Cooperative Oncology Group-performance status of 1 or less, and adequate organ function were included in this study. We diagnosed HCC by blood tests and imaging modalities and histopathological analyses of biopsy specimens for patients with atypical imaging findings. Critical exclusion criteria included no genomic DNA extracted from whole blood, treatment of cases discontinued within 14 days, severe liver failure (Child–Pugh class C), refractory ascites, end-stage kidney disease, or malignancies other than HCC.

### Treatment

Lenvatinib was recommended for patients with advanced BCLC stage C such as portal vein invasion, metastases to distant organs, and for those who were unsuitable and/or refractory to transcatheter arterial therapies. Patients with intermediate BCLC stage B also received systemic lenvatinib therapy at the discretion of their physicians. For patients without risk factors, the recommended dose of lenvatinib was based on body weight: 12 mg/day for patients with a body weight of 60 kg or more and 8 mg/day for patients with a body weight of less than 60 kg. Dose reduction from the initial dose was approved according to the attending physician’s discretion for patients who had risk factors. After the administration of lenvatinib, the dose was changed based on the clinical response and/or the occurrence of severe adverse events. Treatment continued with progressive disease (PD) or until the occurrence of unmanageable adverse events.

### Evaluation for treatment response

We defined tumor response according to the modified Response Evaluation Criteria in Solid Tumors (mRECIST)^43^. Tumor response was visualized by a radiologist using either dynamic computed tomography or dynamic magnetic resonance imaging techniques and was assessed by the attending physician. The target lesion in the mRECIST assessment was defined as the contrast-enhanced portion of the lesion in the arterial phase. For the mRECIST assessment, a complete response (CR) was defined as the disappearance of the target lesion. Partial response (PR) was defined as a reduction of at least 30% of the target lesion size compared to the baseline diameter. PD was defined as an increase of at least 20% of the target lesion size. Stable disease (SD) was defined as any case that does not qualify for either PR or PD.

Objective response was defined as the proportion of patients with the best overall response of confirmed CR or PR. Disease control was defined as the best tumor response of CR, PR, or SD. Patients with an unknown response because of missing data could not be evaluated and were therefore excluded from our analyses. We also recorded the best variation (maximal decrease or minimal increase) in the sum of the most significant lesion dimensions for each patient.

### End-point measurement

To investigate whether or not the NOS3 rs2070744 genotypes can affect the response for lenvatinib treatment in patients with HCC, we examined the influence of the genotypes on the tumor response, PFS, OS, and adverse events. Furthermore, we investigated the impact of the *FGFR* gene on the response in HCC patients treated with lenvatinib. PFS and OS defined as the time from the first dose of lenvatinib to progression or death (for PFS) or death alone (for OS) according to mRECIST. Adverse event assessments were done according to the Common Terminology Criteria for Adverse Events version 4.0 definitions.

### Genomic DNA isolation

Blood samples from the follow-up patients were exclusively collected in EDTA (ethylenediaminetetraacetic acid) tubes. Genomic DNA was extracted from whole blood using an automated MagNA Pure Compact Instrument (Roche Diagnostics GmbH, Mannheim, Germany) with the MagNA Pure Compact Nucleic Acid Isolation Kit I (Roche) following the manufacturer's instructions.

### TaqMan SNP genotyping assays

Genotypes for the NOS3 rs2070744 were determined using the TaqMan SNP Genotyping Assay kits (Applied Biosystems, Foster City, CA, USA). Patients were classified as heterozygotic or homozygotic for each allele. They were categorized as the TT genotype (wild type homozygous mutant), the TC genotype (heterozygous mutant), and the CC genotype (homozygous mutant). As SNPs for the *FGFR* gene, FGFR2 (rs2912791, rs2981429, rs2981582) and FGFR4 (rs351855) were also determined. Chromosomes, positions, and biological effects of the SNPs studied can be found in the Supplementary Table S3 online.

The reaction consisted of 10 μL Master Mix (2 × conc.), 2 μL of the Primer–probe mix (10 × conc.), 6 μL of Water (PCR grade), and 2 μL of genomic DNA sample in a total reaction volume of 20 μL using the FastStart Essential DNA Probes Master kit (Roche Diagnostics GmbH, Mannheim, Germany). The 2-step amplification cycling conditions were: 95 °C for 10 min, followed by 40 cycles of 95 °C for 10 s and 60 °C for 30 s. VIC (victoria) and FAM (fluorescein) fluorescence data were analyzed under Endpoint Genotyping using LightCycler 96 System software version 1.1.0.1320 (Roche Diagnostics GmbH, Mannheim, Germany).

### Statistical analysis

We estimated that this study would require a total of 100 patients by considering loss to follow-up. Variables were examined using Fisher’s exact test or the Chi-square test. The differences between the PFS and OS in the two groups were analyzed by the Kaplan–Meier method and the Log-rank test. Variables identified using the univariate analysis to be significantly associated with PFS and OS were entered into the Cox proportional hazards regression model for multivariate analysis. Univariate and multivariate analyses with logistic regression models were used to calculate the OR at 95% CI to assess the correlation between the response and the NOS3 rs2070744 genotypes for lenvatinib treatment. A two-sided *p* value of less than 0.05 was considered to indicate statistical significance. All analyses were performed using SPSS version 24.0 software (IBM Corporation, Armonk, NY, USA) by the Statista Corporation (Kyoto, Japan) and Stata/MP 15.1 for Mac (StataCorp, College Station, TX, USA).

### Ethics approval

The study was conducted in accordance with the Institutional Review Board for Human Genome Research of the Kitasato University Medical Ethics Organization (KMEO) (Approval Number: KMEO G18-16), the Ethical committee in human genome research of the Yokohama City University (Approval Number: A190725004), and the Tokushukai Group Ethics Committee of Shonan Kamakura General Hospital and Shonan Fujisawa Tokushukai Hospital (Approval Number: TGE01429-024). The results/data/figures in this manuscript have not been published elsewhere, nor are they under consideration (from you or one of your Contributing Authors) by another publisher.

### Consent for publication

The manuscript does not contain clinical studies or patient data.

### Consent to participate

The study was conducted following the Declaration of Helsinki and after obtaining approval of the institutional research ethics committee and written informed consent obtained from all participants.

## Supplementary information

Supplementary Information.

## Data Availability

The technical appendix, statistical code, and dataset are available from the corresponding author.
