# Author Correction: Influence of NOS3 rs2070744 genotypes on hepatocellular carcinoma patients treated with lenvatinib

**DOI:** 10.1038/s41598-021-86457-y

**Published:** 2021-04-14

**Authors:** Shintaro Azuma, Haruki Uojima, Makoto Chuma, Xue Shao, Hisashi Hidaka, Takahide Nakazawa, Masaaki Kondo, Kazushi Numata, Shogo Iwabuchi, Makoto Kako, Shin Maeda, Wasaburo Koizumi, Koichiro Atsuda

**Affiliations:** 1 Department of Pharmacy, Kitasato University Hospital, 1-15-1 Kitasato, Minami-ku, Sagamihara, Kanagawa 252-0375 Japan; 2 Graduate School of Pharmaceutical Sciences, Kitasato University, 5-9-1 Shirokane, Minato-ku, Tokyo, 108-8641 Japan; 3 Department of Gastroenterology, Internal Medicine, Kitasato University School of Medicine, 1-15-1 Kitasato, Minami-ku, Sagamihara, Kanagawa 252-0374 Japan; 4 Gastroenterology Medicine Center, Shonan Kamakura General Hospital, 1370-1 Okamoto, Kamakura, Kanagawa 247-8533 Japan; 5 Gastroenterological Center, Yokohama City University Medical Center, 4-57 Urahune, Minami-ku, Yokohama, Kanagawa 232-0024 Japan; 6 Nakazawa Internal Medicine Clinic, 4-14-18 Sagamidai, Minami-ku, Sagamihara, Kanagawa 252-0321 Japan; 7 Department of Gastroenterology, Yokohama City University Hospital, 3-9 Fukuura, Kanazawa-ku, Yokohama, Kanagawa 236-0004 Japan; 8 Hepato-Biliary-Pancreatic Center, Shonan Fujisawa Tokushukai Hospital, 1-5-1 Tsujidokandai, Fujisawa, Kanagawa 251-0041 Japan; 9 School of Pharmaceutical Sciences, Kitasato University, 5-9-1 Shirokane, Minato-ku, Tokyo, 108-8641 Japan

Correction to: *Scientific Reports* 10.1038/s41598-020-73930-3, published online 13 October 2020

In the original version of this Article, Shintaro Azuma was incorrectly listed as the corresponding author. The correct corresponding author for this Article is Haruki Uojima. Correspondence and request for materials should be addressed to kiruha@kitasato-u.ac.jp.

This error has now been corrected in the HTML and PDF versions of the Article.

