# Supplementary material for: Influence of NOS3 rs2070744 genotypes on hepatocellular carcinoma patients treated with lenvatinib
Source: Sci Rep. 2020 Oct 13;10:17054. doi: 10.1038/s41598-020-73930-3 (PMC7553969; doi:10.1038/s41598-020-73930-3)
Supplement: Supplementary file 1 — Supplementary Information. [file 41598_2020_73930_MOESM1_ESM.pdf]

Influence of NOS3 rs2070744 genotypes on hepatocellular carcinoma patients treated with lenvatinib

Shintaro Azuma,[1, 2] Haruki Uojima,[3, 4] Makoto Chuma,[5] Xue Shao,[3] Hisashi Hidaka,[3] Takahide Nakazawa,[3, 6] Masaaki Kondo,[7] Kazushi Numata,[5] Shogo Iwabuchi,[8] Makoto Kako,[4] Shin Maeda,[7] Wasaburo Koizumi,[3] and Koichiro Atsuda[1, 9]

1. Department of Pharmacy, Kitasato University Hospital, 1-15-1 Kitasato, Minami-ku, Sagamihara, Kanagawa, 252-0375, Japan

2. Graduate School of Pharmaceutical Sciences, Kitasato University, 5-9-1 Shirokane, Minato-ku, Tokyo, 108-8641, Japan

3. Department of Gastroenterology, Internal Medicine, Kitasato University School of Medicine, 1-15-1 Kitasato, Minami-ku, Sagamihara, Kanagawa, 252-0374, Japan

4. Gastroenterology Medicine Center, Shonan Kamakura General Hospital, 1370-1 Okamoto, Kamakura, Kanagawa, 247-8533, Japan

5. Gastroenterological Center, Yokohama City University Medical Center, 4-57 Urahune, Minami-ku, Yokohama, Kanagawa, 232-0024, Japan

6. Nakazawa Internal Medicine Clinic, 4-14-18 Sagamidai, Minami-ku, Sagamihara, Kanagawa, 252-0321, Japan

7. Department of Gastroenterology, Yokohama City University Hospital, 3-9 Fukuura, Kanazawa-ku, Yokohama, Kanagawa, 236-0004, Japan

8. Hepato-Biliary-Pancreatic Center, Shonan Fujisawa Tokushukai Hospital, 1-5-1 Tsujidokandai, Fujisawa, Kanagawa, 251-0041, Japan

9. School of Pharmaceutical Sciences, Kitasato University, 5-9-1 Shirokane, Minato-ku, Tokyo, 108-8641, Japan

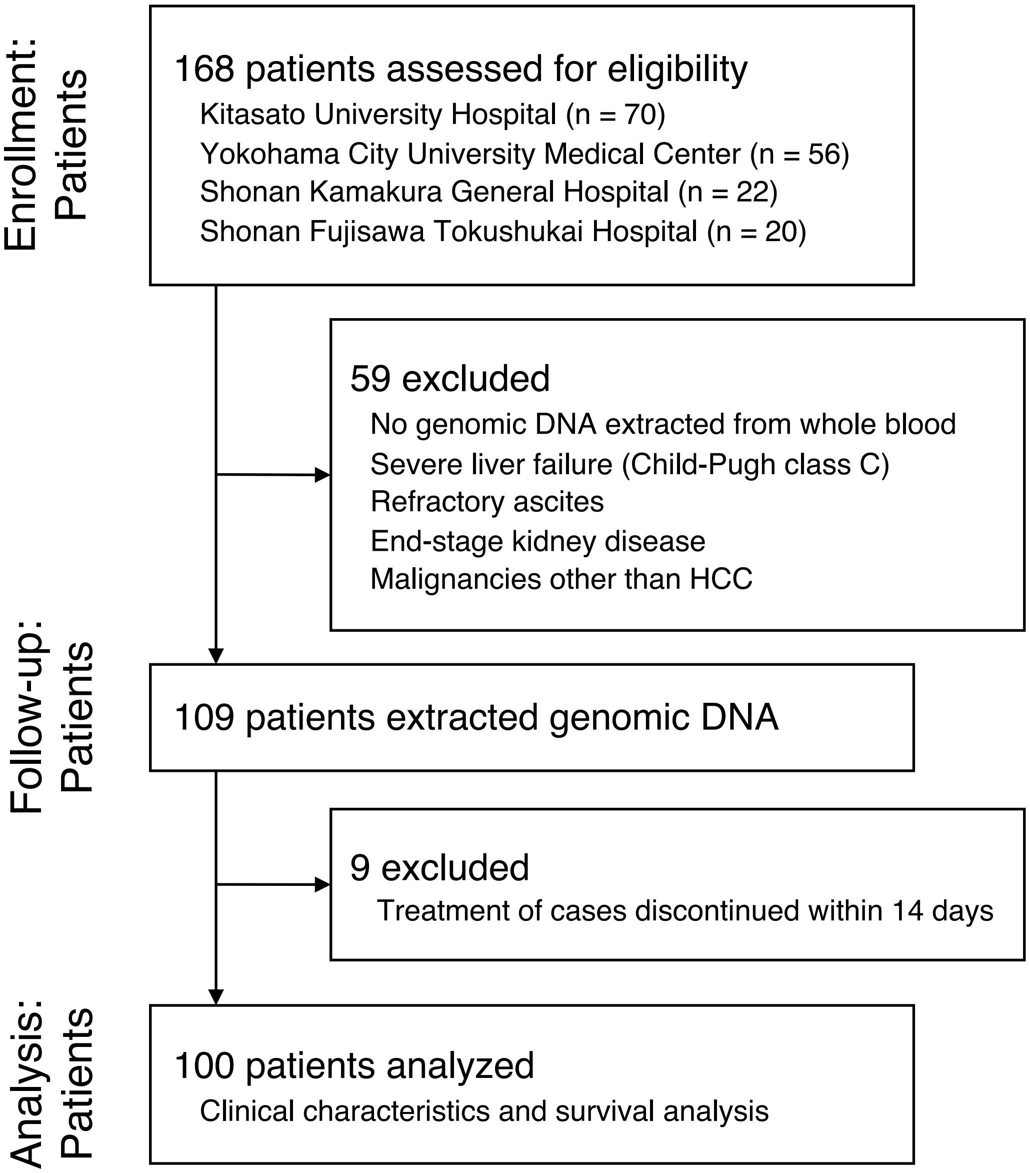

Supplementary Figure S1. Trial profile

Supplementary Table S1. Univariate analysis for baseline prognostic factors for progression-free survival and overall survival

Variable	Category	Progression-free survival		Overall survival	
		HR (95% CI)	*p*	HR (95% CI)	*p*
AFP, ng/mL	< 200 vs. ≥ 200	1.57 (0.95–2.60)	0.081	3.44 (1.47–8.08)	0.004
AFP, ng/mL	< 400 vs. ≥ 400	1.50 (0.90–2.51)	0.119	3.12 (1.35–7.23)	0.008
ALBI grade	Grade 1 vs. Grade ≥ 2	1.35 (0.82–2.22)	0.244	1.52 (0.64–3.60)	0.338
Age, years	< 65 vs. ≥ 65	1.35 (0.76–2.41)	0.311	0.72 (0.29–1.77)	0.473
Age, years	< 75 vs. ≥ 75	1.04 (0.62–1.72)	0.889	1.24 (0.53–2.93)	0.618
BCLC staging system	Stage B vs. Stage C	0.99 (0.61–1.62)	0.975	1.55 (0.66–3.64)	0.314
BMI, kg/m^2	< 21 vs. ≥ 21	1.39 (0.77–2.52)	0.279	2.08 (0.62–7.04)	0.238
Body weight, kg	< 60 vs. ≥ 60	1.19 (0.72–1.97)	0.506	1.63 (0.67–4.02)	0.284
Child-Pugh system	Class A vs. Class B	1.91 (1.06–3.44)	0.031	2.91 (1.22–6.95)	0.016
ECOG-PS	0 vs. 1	0.79 (0.25–2.52)	0.685	N/A	N/A
Etiology of HCC: Non-alcoholic steatohepatitis	no vs. yes	1.04 (0.57–1.88)	0.905	0.79 (0.27–2.33)	0.665
Etiology of HCC: Alcohol use	no vs. yes	0.77 (0.37–1.63)	0.495	3.64 (1.45–9.16)	0.006
Etiology of HCC: Hepatitis B	no vs. yes	1.64 (0.90–2.98)	0.106	0.68 (0.20–2.31)	0.542
Etiology of HCC: Hepatitis C	no vs. yes	0.66 (0.39–1.12)	0.121	0.57 (0.22–1.46)	0.242
Extrahepatic spread present	no vs. yes	0.67 (0.38–1.18)	0.163	0.87 (0.35–2.17)	0.768
FGFR4 rs351855 genotype	CT/TT vs. CC	1.17 (0.70–1.94)	0.553	1.02 (0.43–2.44)	0.963
Hepatic ascites	no vs. yes	1.60 (0.85–3.01)	0.146	4.40 (1.82–10.62)	0.001
Hepatic encephalopathy	no vs. yes	1.70 (0.68–4.25)	0.258	1.77 (0.41–7.58)	0.443
Intrahepatic lesion	no vs. yes	0.33 (0.10–1.09)	0.069	N/A	N/A
Macroscopic PVI, extrahepatic spread, or both, present	no vs. yes	0.90 (0.55–1.50)	0.699	1.50 (0.65–3.49)	0.345
Macroscopic PVI present	no vs. yes	1.71 (0.93–3.14)	0.084	3.89 (1.60–9.49)	0.003
Maximum tumor size, cm	≤ 5 vs. > 5	1.26 (0.74–2.14)	0.397	2.70 (1.15–6.33)	0.022
NOS3 rs2070744 + FGFR4 rs351855 genotypes	Others vs. TC/CC + CT/TT	2.56 (1.28–5.09)	0.008	3.36 (1.21–9.29)	0.020
NOS3 rs2070744 genotype	TC/CC vs. TT	0.60 (0.34–1.05)	0.073	0.46 (0.19–1.11)	0.083
Number of tumors	< 4 vs. ≥ 4	0.91 (0.56–1.48)	0.701	1.26 (0.54–2.93)	0.588
PIVKA-II, mAU/mL	< 100 vs. ≥ 100	1.00 (0.57–1.77)	0.988	1.22 (0.45–3.31)	0.696
Portal vein invasion	VP 0–3 vs. VP ≥ 4	1.13 (0.45–2.85)	0.789	1.44 (0.33–6.24)	0.622
RDI at 56 days, %	≥ 70 vs. < 70	1.09 (0.65–1.82)	0.745	1.28 (0.53–3.10)	0.588
RDI at 56 days, %	≥ 75 vs. < 75	1.06 (0.64–1.77)	0.818	1.09 (0.45–2.64)	0.847
RDI at 56 days, %	≥ 80 vs. < 80	1.00 (0.60–1.66)	0.988	0.83 (0.34–2.01)	0.682
Sex	female vs. male	1.10 (0.64–1.89)	0.722	2.09 (0.71–6.17)	0.184
Tumor liver occupying rate, %	< 50 vs. ≥ 50	1.03 (0.37–2.87)	0.948	1.92 (0.45–8.26)	0.381
UICC-TNM staging system	I and II vs. III and IV	0.96 (0.54–1.72)	0.899	1.19 (0.40–3.53)	0.750
Up-to-seven criteria	within vs. beyond	1.02 (0.62–1.68)	0.945	1.68 (0.73–3.91)	0.224

AFP α-fetoprotein, *ALBI* albumin-bilirubin, *BCLC* Barcelona Clinic Liver Cancer, *BMI* body mass index, *BSC* best supportive care, *CI* confidence interval, *ECOG-PS* Eastern Cooperative Oncology Group-Performance Status, *FGFR4* fibroblast growth factor receptor 4, *HCC* hepatocellular carcinoma, *HR* hazard ratio, *N/A* not available, *NOS3* nitric oxide synthase 3, *PIVKA-Ⅱ* protein induced by vitamin K absence or antagonist Ⅱ, *PVI* portal vein invasion, *RDI* relative dose intensity, *UICC-TNM* Union for International Cancer Control-Tumor, Nodes, Metastasis.

Supplementary Table S2. Treatment-related adverse events occurring in either group

Adverse event, n (%)	N	All (N = 100)		*NOS3 rs2070744*				*FGFR4 rs351855*			
				TT (n = 76)		TC + CC (n = 24)		CC (n = 37)		CT + TT (n = 63)	
		Grade ≤ 2	Grade 3	Grade ≤ 2	Grade 3	Grade ≤ 2	Grade 3	Grade ≤ 2	Grade 3	Grade ≤ 2	Grade 3
Any adverse event	76	73 (96)	43 (57)	55 (72)	33 (43)	18 (75)	10 (42)	28 (76)	16 (43)	45 (71)	27 (43)
Anorexia	59	42 (71)	17 (29)	30 (39)	13 (17)	12 (50)	4 (17)	18 (49)	7 (19)	24 (38)	10 (16)
Fatigue	55	54 (98)	1 (2)	39 (51)	1 (1)	15 (63)	0	22 (59)	0	32 (48)	1 (2)
PPE	47	45 (96)	2 (4)	31 (41)	1 (1)	14 (58)	1 (4)	19 (51)	0	26 (32)	2 (3)
Abdominal pain	41	38 (93)	3 (7)	26 (34)	3 (4)	12 (50)	0	13 (35)	1 (3)	25 (40)	2 (3)
Diarrhea	41	37 (90)	4 (10)	25 (33)	3 (4)	12 (50)	1 (4)	15 (41)	3 (8)	22 (35)	1 (2)
Dysgeusia	36	36 (100)	0	26 (34)	0	10 (42)	0	13 (35)	0	23 (37)	0
Edema	36	34 (94)	2 (6)	27 (36)	1 (1)	7 (29)	1 (4)	13 (35)	0	21 (33)	2 (3)
Anxiety	35	32 (91)	3 (9)	23 (30)	3 (4)	9 (38)	0	9 (24)	2 (5)	23 (37)	1 (2)
Hypertension	35	28 (80)	7 (20)	21 (28)	6 (8)	7 (29)	1 (4)	12 (32)	3 (8)	16 (25)	4 (6)
Constipation	29	25 (86)	4 (14)	19 (25)	4 (5)	6 (25)	0	6 (16)	3 (8)	19 (30)	1 (2)
Nausea/vomiting	27	26 (96)	1 (4)	21 (28)	1 (1)	5 (21)	0	13 (35)	0	13 (21)	1 (2)
Palpitations	27	25 (93)	2 (7)	19 (25)	2 (3)	6 (25)	0	9 (24)	2 (5)	16 (25)	0
Rash	26	26 (100)	0	17 (22)	0	9 (38)	0	10 (27)	0	16 (25)	0
Mucositis oral	24	22 (92)	2 (8)	13 (17)	2 (3)	9 (38)	0	9 (24)	1 (3)	13 (21)	1 (2)
Proteinuria	20	13 (65)	7 (35)	9 (12)	6 (8)	4 (17)	1 (4)	7 (19)	1 (3)	6 (10)	6 (10)
Peripheral neuropathy	19	19 (100)	0	14 (18)	0	5 (21)	0	6 (16)	0	13 (21)	0
Nail change	13	13 (100)	0	11 (14)	0	2 (8)	0	3 (8)	0	10 (16)	0
Eye disorders	12	12 (100)	0	8 (11)	0	4 (17)	0	4 (11)	0	8 (13)	0
Hypothyroidism	11	11 (100)	0	9 (12)	0	2 (8)	0	2 (5)	0	9 (14)	0
Paronychia	7	7 (100)	0	6 (8)	0	1 (4)	0	0	0	7 (11)	0
Ascites	6	2 (33)	4 (67)	2 (3)	4 (5)	0	0	1 (3)	3 (8)	1 (2)	1 (2)
Fever	6	6 (100)	0	3 (4)	0	3 (13)	0	4 (11)	0	2 (3)	0
Platelet count decreased	6	4 (67)	2 (33)	3 (4)	2 (3)	1 (4)	0	0	0	4 (6)	2 (3)

FGFR4 fibroblast growth factor receptor 4, *NOS3* nitric oxide synthase 3, *PPE* palmar-plantar erythrodysesthesia.

Supplementary Table S3. Chromosome, positions and biological effect of studied single-nucleotide polymorphisms

Gene	Gene name	SNP	TaqMan catalog number	Chr	Position	Functional consequence	Amino acid change
NOS3	Nitric oxide synthase 3	rs2070744	C_15903863_10	7	150992991	Intron variant	N/A
FGFR2	Fibroblast growth factor receptor 2	rs2912791	C_16166432_10	10	121559447	Intron variant	N/A
		rs2981429	C_2917349_10	10	121552460	Intron variant	N/A
		rs2981582	C_2917302_10	10	121592803	Intron variant	N/A
FGFR4	Fibroblast growth factor receptor 4	rs351855	C_3166614_10	5	177093242	Missense variant	Gly388Arg

Arg arginine, *Chr* chromosome, *Gly* glycine, *N/A* not available, *SNP* single-nucleotide polymorphism.
